# Non-albuminuric Diabetic Kidney Disease Phenotype: Beyond Albuminuria

**DOI:** 10.17925/EE.2022.18.2.102

**Published:** 2022-11-16

**Authors:** Luis D’Marco, Xavier Guerra-Torres, Iris Viejo, Luis Lopez-Romero, Alejandra Yugueros, Valmore Bermídez

**Affiliations:** 1. Universidad Cardenal Herrera-CEU, CEU Universities, Valencia, Spain; 2. Hospital General Universitario de Valencia, Valencia, Spain; 3. Hospital Universitario Príncipe de Asturias, Madrid, Spain; 4. Nephrology Department, Hospital Universitari i Politècnic La Fe, Valencia, Spain; 5. Internal Medicine Department, Hospital Arnau de Vilanova, Valencia, Spain; 6. Universidad Simèn Bolívar, Facultad de Ciencias de la Salud, Barranquilla, Colombia

**Keywords:** Diabetes, albuminuria, chronic kidney disease, cardiovascular disease

## Abstract

Diabetic kidney disease (DKD) is the leading cause of chronic and end-stage kidney disease worldwide. Its pathogenic mechanism is complex, and it can affect the entire structures of the kidneys such as the glomerulus, tubules and interstitium. Currently, the urinary albumin excretion rate and the estimated glomerular filtration rate are widely accepted as diagnostic criteria. However, some studies have reported a different or non-classical clinical course of DKD, with some patients showing declined kidney function with normal levels of albuminuria, known as the ‘non-albuminuric DKD’ phenotype. The pathogenesis of this phenotype remains unclear, but some clinical and pathological features have been postulated. This review explores the evidence regarding this topic.

Diabetic kidney disease (DKD) is the leading cause of chronic kidney disease (CKD) and end-stage kidney disease (ESKD) worldwide.^1,2^ For this reason, early diagnosis and treatment are relevant to prevent the progression of this disease. Currently, the urinary albumin excretion rate and the estimated glomerular filtration rate (eGFR) are widely accepted as diagnosis criteria, and the presence of microalbuminuria has been recommended as the first clinical sign of DKD.^3^

Beyond this classical clinical course, some studies report a non-classical clinical course of DKD, where kidney function declines but albuminuria levels are normal. This group of patients is recognized as having the ‘non-albuminuric DKD’ (NA-DKD) phenotype.^4–9^ Recent evidence has shown that around 20–40% of patients with type 2 diabetes mellitus (T2DM) have declining kidney function without the presence of albuminuria.^10,11^ Currently, the pathogenesis of NA-DKD remains unclear, but some clinical and pathological features of this phenotype have been postulated. Here, we review the evidence regarding this topic.

## Diabetic kidney disease

### Epidemiology

The rapid increase in the prevalence and incidence of diabetes has led to a rising prevalence of diabetes-related complications. DKD, a common microvascular complication of diabetes, has become a worldwide public health problem.^1^

The reported prevalence of NA-DKD varies between regions. The Third National Health and Nutrition Examination Survey (NHANES III) data indicated that albuminuria and retinopathy were both absent in 30% of adults with T2DM and CKD among US civilians.^12^ In the UK Prospective Diabetes Study, which followed 9,063 patients with T2DM for 15 years, 61% of patients who developed CKD did not have albuminuria during the follow-up period.^13^ In Korea and Japan, the reported prevalence of NA-DKD among patients with T2DM is 29.1% and 52.7%, respectively.^14,15^ According to other studies, approximately 9.3–71.1% of patients with an eGFR <60 mL/min/1.73 m^2^ had normal albuminuria.^16^ Finally, a Chinese study showed that the prevalence of normal albuminuria but reduced eGFR was 8.3% in all patients with diabetes, and 63.3% in those who had an eGFR decline.^17^

Currently, the causes of the increased prevalence of NA-CKD and NA-DKD could be related to the widespread use of renin-angiotensin system blockers and their combinations with the new classes of hypoglycaemic agents, including sodium–glucose cotransporter 2 (SGLT2) inhibitors and glucagon-like peptide 1 receptor agonists (GLP1-RAs). This situation would increase the proportion of NA-CKD among subjects with T2DM and renal failure.

Regarding cardiovascular outcomes, patients with albuminuric DKD had the highest risk of adverse outcomes compared with patients without DKD. However, results were conflicting when comparing patients with albuminuric versus NA-DKD.^18^

Therefore, albuminuric and NA-DKD patterns significantly differ in their natural history and outcomes. Although NA-DKD is a more favourable condition regarding ESKD risk, it is associated with cardiovascular disease and its risk factors. Both increased albuminuria and reduced eGFR are predictors of ESKD and cardiovascular events in patients with T2DM.^6^

### Trajectories

Although there are few studies addressing this atypical presentation of DKD, factors associated with risk for NA-DKD include female sex, hypertension, active smoking, the absence of diabetic retinopathy and the use of renin–angiotensin–aldosterone system (RAAS) inhibitors.^15,17,19^ In classical DKD, retinopathy usually precedes albuminuria as a microvascular complication; however, without albuminuria, the presence of retinopathy supports a pathway of DKD progression distinct from that of the classical phenotype.^6,9^ In patients without diabetes with moderate CKD, any type of proteinuria (albuminuria and non-albuminuric proteinuria) is present in around 22%.^20^ As in NA-DKD, tubular damage is associated with the presence of inflammatory markers in CKD.^20^

Of note, NA-DKD appears to have a better clinical course than classical DKD, with a slower decline in eGFR and a lower risk of CKD progression.^6^ However, some intercurrent factors are associated with worse clinical outcomes in patients with NA-DKD.^6^ Factors such as increasing age, macrovascular complications and interstitial nephritis could modify the course of the disease.^17^ Overall, NA-DKD tends to be associated with more advanced tubulointerstitial and vascular lesions, but milder typical glomerular damage compared with patients with albuminuric DKD.^17^ Further exploration of the factors other than albuminuria that contribute to disease progression will help elucidate the mechanisms of NA-DKD.

In normal ageing, the annual rate of eGFR decline is estimated to be <1 mL/min/1.73 m^2^; however, in patients with T2DM, the median eGFR slope varied from -1.5 mL/min/1.73 m^2^/year to -4.0 mL/min/1.73 m^2^/ year.^6,21^ In healthy individuals, this decline is attributed mainly to age-related structural changes, such as nephron loss. On the contrary, patients with NA-DKD might have different basal conditions, such as interstitial nephritis or nephrosclerosis, leading to eGFR decline.^21^ In patients with T1DM with normal albuminuria, an investigation found that 10% (N=286) experienced a progressive decline in eGFR (≥3.3%/year) before the onset of microalbuminuria.^22^

### Histological evidence

Renal biopsy in patients with diabetes is usually performed in those with significant renal manifestations such as severe proteinuria, microscopic haematuria, rapid and unexplained worsening of kidney function, or a decrease of more than 30% in eGFR after starting RAAS inhibitors.^6^ Currently, renal biopsy is not routinely indicated in these patients; however, it is still the gold standard for diagnosing diabetic nephropathy (DN), and recent evidence favours determining the histology of renal disease in patients with diabetes.^23,24^ Therefore, very few studies have analysed renal histological alterations in the early stages of diabetes.^25^

A recent study from necropsies found that a significant percentage of patients with diabetes without early clinical signs of DKD such as microalbuminuria already show moderate to severe histological involvement related to DN; however, comorbidities and renal progression factors did not significantly differ from patients with albuminuria.^26^ These findings suggest early renal involvement in patients with NA-DKD.

The tubulointerstitium is composed of tubular epithelium, vascular structures and interstitium, and accounts for 90% of renal tissue.^27^ The classical pathological interstitial changes in DN include tubular basement membrane thickening, interstitial fibrosis, tubular atrophy and arteriosclerosis.^28^ A study found that interstitial expansion was independently associated with reduced kidney function in T1DM.^29^ Another investigation in patients with DN showed that interstitial fibrosis contributes to further kidney function decline compared with glomerular injury, and this decline was independent of albuminuria.^30^ This suggests that eGFR decline is partly related to interstitial injury in T2DM. Ekinci et al. found that three of eight (37.5%) patients with NA-DKD had interstitial and vascular lesions by renal biopsy, and only 1 in 23 (4.3%) patients with albuminuric DKD had interstitial and vascular lesions.^31^

Beyond the histological evidence, the prevailing underlying pathology of NA-DKD might be macroangiopathy instead of microangiopathy. Moreover, some findings suggest that renal impairment in NA-DKD is not mainly caused by hyperglycaemia or microangiopathy, and that genetic susceptibility, ageing and arteriosclerosis might contribute more to this specific phenotype.^32^ In accordance, investigations reported that eGFR decline without albuminuria was associated with age-dependent arterial stiffness in patients with and without diabetes.^33,34^ This may explain why other renal conditions such as hypertensive nephropathy or age-related nephrosclerosis have similar phenotypes.

### Novel biomarkers

Conventional approaches to evaluating and monitoring the early stages of DKD have focused on changes in the glomerulus. However, the current standard screening tools, albuminuria, and eGFR are especially insufficient in detecting early tubular and other kidney damage.^35^ Therefore, many tubular biomarkers have been suggested.^35^

As albuminuria is a marker for glomerular damage, biomarkers that reflect other pathological mechanisms of DKD, such as tubular injury, would be acceptable complementary DKD markers to albuminuria. Tubular injury markers such as α1-microglobulin (A1M), liver-type fatty acid-binding protein (L-FABP), N-acetyl-β-D-glycosaminidase (NAG), urinary immunoglobulin G and M, and kidney injury molecule-1 (KIM-1) are associated with DKD.^36^

More recently, proteomic and metabonomic analyses have shown promise in evaluating the NA-DKD phenotype. Metabonomic assessment is a relatively new biological approach that provides global metabolic information in biological samples. Hippurate and allantoin are associated with reduced eGFR and are potential markers of nephrotoxicity and tubular dysfunction.^37^ Proteomics is a large-scale experimental analysis of proteins through protein purification and mass spectrometry. Proteomic studies have revealed collagen fragments as a potential biomarker of DKD, even 3–5 years before the onset of microalbuminuria in patients with diabetes.^38^

### Therapeutic tools

**Figure 1:**
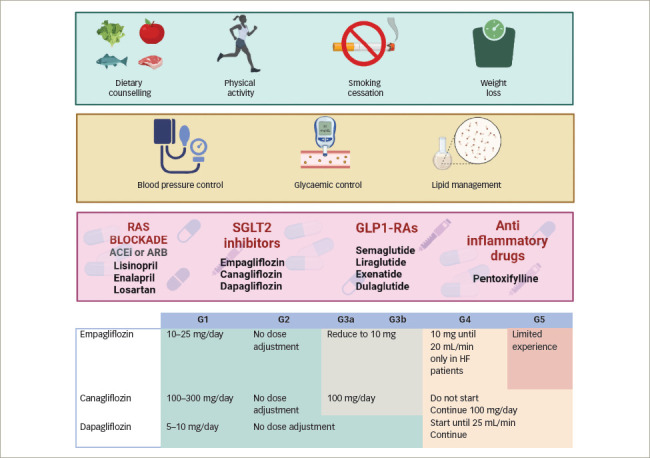
Multifactorial intervention to target kidney disease progression and cardiovascular disease risk factors in patients with diabetes

Optimal management of NA-DKD is complex and not entirely known. Nonetheless, multifactorial intervention is needed to target kidney disease progression and cardiovascular disease risk factors. Initial therapy consists of lifestyle modification, including dietary counselling, physical activity and smoking cessation support. Furthermore, controlling hypertension, dyslipidaemia and hyperglycaemia is essential, as in patients with albuminuric DKD (*Figure 1*).^39^

Recently, first-line drug therapy proposed by the Kidney Disease Improving Global Outcomes (KDIGO) 2020 clinical practice guideline for diabetes management in CKD includes RAAS blockade, metformin, SGLT2 inhibitors and statins.^40^ Angiotensin-converting enzyme inhibitors and angiotensin II receptor blockers (ARBs) are widely recommended in patients with DKD and albuminuria. However, less evidence exists for patients with NA-DKD. Some studies have found that lisinopril, enalapril and losartan are effective at reducing progression of kidney disease and controlling blood pressure in patients with NA-DKD.^41,42^

*ACEi = angiotensin-converting enzyme inhibitor; ARB = angiotensin II receptor blocker; G = grade of kidney disease; GLP1 = glucagon-like peptide 1; HF = heart failure;*

*RA = receptor agonist; RAS = renin–angiotensin system; RAAS = renin–angiotensin–aldosterone system; SGLT2 = sodium–glucose cotransporter 2.*

SGLT2 inhibitors have several cardiovascular and renal benefits in patients with diabetes.^43–46^ In addition to reducing blood pressure and improving metabolic parameters such as glycated haemoglobin (HbA1c) and weight, SGLT2 inhibitors provide renal protection by correcting glomerular hyperfiltration, reducing albuminuria, improving kidney hypoxia and reducing proinflammatory and profibrotic pathways.^47^ Although there is no subgroup analysis of albuminuria in the pivotal studies of the different SGLT2 inhibitors, the EMPA-REG OUTCOME,^46^ CANVAS^44^ and DECLARE-TIMI 58^43^ studies analysed empagliflozin, canagliflozin and dapagliflozin, respectively, and included both non-albuminuric and albuminuric patients. Of note, DECLARE-TIMI 58 demonstrated the effect of dapagliflozin in earlier stages of kidney disease, as 65% of the patients showed no expression of any renal markers (eGFR <60 mL/min/1.73 m^2^, micro- or macroalbuminuria; 93% showed eGFR >60 mL/min/1.73 m^2^ and 69% showed normal albuminuria) and 59% of patients had no previous cardiovascular disease.^43^ Interestingly, dapagliflozin seems to prevent and reduce DKD progression compared with placebo in T2DM patients with or without established atherosclerotic cardiovascular disease, most of whom had preserved renal function.^43,44^ Although the possible mechanisms of benefit were multiple and incompletely understood, in most cases this benefit, seen in all SGLT2 inhibitors, has been independently associated with glycaemic control.^48^ On the contrary, one exploratory analysis of the EMPA-REG OUTCOME study found no significant between-group difference in the rate of albuminuria.^49^ New clinical trials explicitly testing the effect of SGLT2 inhibition in patients with NA-DKD are needed.

The next step of the therapy proposed by the KDIGO guidelines is GLP1-RAs and non-steroidal mineralocorticoid receptor antagonists (NS-MRAs).^50–52^ However, in pivotal studies for GLP1-RAs, baseline albuminuria levels were not specified, although a trial testing semaglutide did not exclude normoalbuminuric patients.^53^ GLP1-RAs in patients with T2DM and CKD are safe and well tolerated, and significantly improve cardiovascular and renal variables.^50^ On the other hand, in NS-MRA studies, there were no patients with NA-DKD;^49^ thus, current recommendations are unavailable for these patients.

Finally, given the inflammatory activities associated with diabetes, some anti-inflammatory drugs, such as the tumour necrosis factor-α inhibitor pentoxifylline, decrease or stabilize the progression of DN with additional renoprotective effects.^54,55^ Because patients with NA-DKD have elevated levels of tumour necrosis factor-α and other cytokines, they could respond to pentoxifylline. Further studies investigating this and other anti-inflammatory drugs in patients with NA-DKD are needed.

## Conclusion

Diagnosing and managing the NA-DKD phenotype represents a real challenge for the nephrology community, as more accurate diagnosis methods are needed alongside albuminuria. Detecting biomarkers early may help to avoid DKD progression. The rapid rise of new agents such as SGLT2 inhibitors, GLP1-RAs, NS-MRAs and their combinations may contribute to improving the outcomes of patients with NA-DKD, though further study is needed.
